# Parallel associations between cognitive and neuropsychiatric symptom domains in older adults

**DOI:** 10.1093/geroni/igaf122.3634

**Published:** 2025-12-31

**Authors:** Alyssa De Vito, Adea Rich, Megan Barker, Zachary Kunicki

**Affiliations:** Brown University, Providence, Rhode Island, United States; Columbia University, New York, New York, United States; Brown University, Providence, Rhode Island, United States; Warren Alpert Medical of Brown University, Providence, Rhode Island, United States

## Abstract

Despite the high prevalence of neuropsychiatric symptoms (NPS) in those with Alzheimer’s disease and Alzheimer’s disease-related dementias (AD/ADRD), longitudinal research examining the co-occurrence of NPS and cognition has been underexplored, especially during earlier stages of the disease. Our study aimed to investigate the co-occurring trajectories between specific domains of NPS and cognition in those with preclinical or prodromal AD/ADRD. Participants were 1,255 individuals from the Alzheimer’s Disease Neuroimaging Initiative (ADNI)-2 and ADNI-3 cohorts who were cognitively normal (CN) or had mild cognitive impairment (MCI) at baseline. This cohort was evenly split by sex and was predominantly White and non-Hispanic. Parallel process models were used to examine how a change in cognition was associated with a change in NPS as measured by the Neuropsychiatric Inventory (NPI) over 48 months. We ran 9 models total to examine relationships between 3 cognitive domains (memory, executive function, and language) and 3 NPS domains (impulse dyscontrol, mood, and somatic disturbance). Cognitive domain and NPS slopes were negatively correlated in each model, such that as cognition decreased, NPS increased. Follow-up testing using Steiger’s z-test to compare the correlations across domains suggested no significant differences in the strength of the relationship between mood or somatic disturbance with cognition across domains. However, impulse dyscontrol had a significantly stronger relationship with language than with other cognitive domains. These findings extend prior cross-sectional work that found communication-impulse dyscontrol behavior (e.g., aggression) associations to demonstrate strong co-occurrence longitudinally in those with early-stage AD/ADRD.

